# Interpretable machine learning for coronary heart disease risk stratification in patients with carotid atherosclerosis: A retrospective cross-sectional study

**DOI:** 10.1097/MD.0000000000047203

**Published:** 2026-01-16

**Authors:** Lei Zhang, Mengke Lyu, Mingyuan Du, Yizhuo Li, Haifeng Yan, Xiaohui Li, Wenshuang Niu, Lizhi Pang

**Affiliations:** a Heart Center, The First Affiliated Hospital of Henan University of Chinese Medicine, National Regional (TCM) Cardiovascular Diagnosis and Treatment Center, Zhengzhou, Henan, China; b The First Affiliated Hospital of Henan University of Chinese Medicine, Zhengzhou, Henan, China; c The Fifth Clinical Medical College of Henan University of Traditional Chinese Medicine, Zhengzhou, Henan, China; d The First Clinical Medical College of Zhengzhou University, Zhengzhou, Henan, China.

**Keywords:** carotid atherosclerosis, coronary heart disease, logistic regression, machine learning, predictive model, SHAP

## Abstract

This study aimed to develop and validate a machine learning model for risk stratification of coronary heart disease (CHD) in patients with carotid atherosclerosis, with CHD presence/absence defined as the target outcome variable. A retrospective analysis was conducted on 442 patients diagnosed with carotid atherosclerosis at a tertiary hospital in China between January 1, 2022, and June 20, 2025. Patients were divided into CHD and non-CHD groups based on clinical outcomes. Data encompassing demographics, laboratory results, and vascular imaging findings were collected. Feature selection involved logistic regression (LR), identifying 5 key predictors: age, diabetes, hyperlipidemia, transient ischemic attack (TIA), and the presence of carotid atherosclerotic plaque. Seven machine learning algorithms (LR, XGBoost, LightGBM, random forest, K-nearest neighbors, support vector machine, and stacking ensemble) were trained and evaluated. Model performance was assessed using 10-fold cross-validation, with metrics including area under the curve, accuracy, sensitivity, specificity, and F1 score. Model interpretability was evaluated using Shapley Additive Explanations, while clinical utility was determined through calibration and decision curve analysis. All models demonstrated satisfactory performance, with the LR model achieving the highest area under the curve of 0.838 on the testing set, indicating balanced sensitivity and specificity. Shapley Additive Explanations analysis identified carotid plaque and TIA as the most influential predictors. Calibration and decision curve analysis curves indicated strong agreement between predicted and observed risks, leading to a significant clinical net benefit. An interpretable LR model incorporating age, diabetes, hyperlipidemia, TIA, and carotid plaque enables reliable CHD risk stratification among patients with carotid atherosclerosis. This model serves as a practical, explainable tool for individualized risk assessment and early clinical decision support in this high-risk population.

## 1. Introduction

Coronary heart disease (CHD) remains a significant global health concern due to its high morbidity and mortality rates, particularly with the aging population and the rising prevalence of metabolic disorders.^[1]^ In China, >11.39 million individuals are affected by CHD, with a troubling trend of earlier onset among younger age groups, as noted in the 2022 China Cardiovascular Health and Disease Report.^[2]^ Atherosclerosis, the primary pathological process underlying CHD, is characterized by lipid accumulation, chronic inflammation, and endothelial dysfunction.^[3]^ Carotid atherosclerotic plaque, commonly known as carotid plaque, is widely recognized as a key indicator of systemic vascular pathology and a strong predictor of future cardiovascular events such as myocardial infarction and stroke.^[4]^ Early identification of subclinical atherosclerosis is crucial for prompt intervention and effective risk mitigation.

Several prediction models, including the Framingham Risk Score (FRS) and atherosclerotic cardiovascular disease (ASCVD) risk estimator (both widely recommended in the 2019 American College of Cardiology/American Heart Association [ACC/AHA] Guideline on the Primary Prevention of Cardiovascular Disease^[5]^) have been created to evaluate CHD risk. Nevertheless, these standard tools are predominantly founded on generalized Western populations and might not be ideal for subgroups with distinct vascular traits, like individuals with confirmed carotid atherosclerosis.^[6]^ Additionally, conventional models frequently rely on linear assumptions and incorporate only a restricted set of variables, possibly neglecting the complex, nonlinear interplays among metabolic, vascular, and lifestyle elements. Notably, conventional risk scores such as the FRS and ASCVD estimator exhibit poor predictive performance in patients with carotid atherosclerosis, with area under the curve (AUC) values close to 0.5 in our study cohort (Fig. S1, Supplemental Digital Content, https://links.lww.com/MD/R154). These findings underscore the inadequacy of current tools and highlight the need for a tailored, interpretable model for this high-risk subgroup.

Recent advances in artificial intelligence, specifically in machine learning (ML), have transformed clinical risk prediction by enabling the analysis of intricate, multidimensional datasets.^[7]^ ML algorithms have shown enhanced efficacy in disease classification and prognosis compared to conventional statistical approaches, especially in situations with varied patient characteristics.^[8,9]^ However, there is a lack of studies employing ML methods to forecast CHD risk in individuals with carotid atherosclerosis, despite their elevated baseline risk and the potential for early preventive measures.

This study aimed to develop and validate a ML model to predict the presence of CHD in patients with carotid atherosclerosis, with CHD presence/absence defined as the target outcome variable. A retrospective dataset comprising 442 patients was employed to amalgamate demographic, biochemical, and vascular imaging data for the development of 7 ML models, encompassing logistic regression (LR), XGBoost, LightGBM, random forest, support vector machine (SVM), K-nearest neighbors (KNN), and a stacking ensemble. The performance of each model was assessed through 10-fold cross-validation, with the most effective algorithm further elucidated using SHAP (Shapley Additive Explanations) to ensure transparency and clinical interpretability. This study introduces a robust and interpretable methodology for forecasting CHD risk in a high-risk vascular cohort, thereby advancing precision cardiovascular prevention and optimizing care delivery.

## 2. Materials and methods

### 2.1. Study subjects

This retrospective cross-sectional study analyzed 442 adult patients aged 18 to 80 with carotid atherosclerosis, diagnosed by ultrasound or computed tomography angiography (CTA), who were treated at the First Affiliated Hospital of Henan University of Chinese Medicine between January 1, 2022, and June 20, 2025. All participants had confirmed carotid atherosclerosis at baseline. CHD status was determined according to standardized diagnostic criteria (see Section 1.3) and classified as present (CHD group) or absent (non-CHD group) at the time of enrollment. To minimize temporal ambiguity, patients with a new CHD diagnosis within 6 months after enrollment were excluded from the study.

*Justification of sample size*: The final sample size of 442 patients was determined based on the total number of eligible cases identified during the study period according to predefined inclusion and exclusion criteria. Due to the retrospective nature of the study, no a priori sample size calculation was performed. However, a post hoc power analysis utilizing G*Power (version 3.1; Heinrich-Heine-Universität Düsseldorf, Düsseldorf, Germany) revealed that a minimum of 192 participants (68 in the CHD group and 124 in the non-CHD group) would be adequate to detect a moderate difference in CHD prevalence (25% vs 45%) with 80% power at a two-sided significance level of 0.05. Additionally, given that the model included 5 predictive features, the sample size met the recommended minimum of 10 to 15 outcome events per predictor. With 156 CHD cases, the model achieved an average of 31.2 events per predictor, ensuring sufficient statistical power and robustness for the development and validation of ML models.

Participants were divided into 2 cohorts based on clinical diagnosis of CHD: the CHD group and the non-CHD group. CHD diagnosis adhered to established criteria, including stable angina or Acute Coronary Syndrome, consistent with the 2023 European Society of Cardiology Guidelines for Acute Coronary Syndrome Management^[10]^ and the 2021 ACC/AHA Guideline for the Evaluation and Diagnosis of Chest Pain.^[11]^ All participants underwent comprehensive assessment, including demographic, laboratory, and imaging evaluations. Exclusion criteria encompassed congenital heart disease, non-atherosclerotic cardiac conditions (e.g., myocarditis), active malignancy, severe psychiatric disorders, pregnancy or lactation, and involvement in other clinical trials within the preceding 3 months. The study protocol was approved by the Ethics Committee of the First Affiliated Hospital of Henan University of Chinese Medicine (2024HL-159-01).

### 2.2. Data collection and feature variables

Clinical data were extracted from the hospital’s electronic medical records and laboratory information systems and meticulously curated by 2 trained research physicians using a standardized case report form. The dataset comprised demographic variables (age, sex, education level, body mass index), clinical history (hypertension, diabetes mellitus, hyperlipidemia, transient ischemic attack, smoking status, alcohol intake), laboratory parameters (complete blood count, lipid profile, coagulation markers, renal function, fasting glucose), imaging findings (carotid plaque presence detected via Doppler ultrasound or CTA), and data quality assurance protocols. To uphold data accuracy, all variables were validated by cross-referencing with primary reports, and any missing or outlier values were managed according to predefined quality control procedures.

### 2.3. Outcome definition

For the predictive model, the target outcome variable was the presence or absence of CHD.

CHD was defined according to standardized clinical criteria^[10–12]^ and classified into 3 subcategories:

(1) prior myocardial infarction or coronary revascularization;
(2) angiographically confirmed coronary stenosis ≥50% with angina symptoms;
(3) high-risk coronary stenosis confirmed by coronary CT angiography with objective evidence of myocardial ischemia.

For analysis, these subcategories were collectively considered as a binary outcome (CHD presence vs absence), consistent with established cardiovascular risk prediction models.

## 3. Statistical and ML methods

### 3.1. Feature selection

Statistical analyses were performed using SPSS 27 (IBM, Armonk), R 4.5.0 (The R Foundation for Statistical Computing, Vienna, Austria), Python 3.13.3 (Python Software Foundation, Beaverton) . Quantitative variables were expressed as mean ± standard deviation and compared using independent *t* tests or Mann–Whitney *U* tests, depending on normality. Categorical variables were compared using the Chi-square test or Fisher exact test.

A total of 32 candidate variables were initially considered for model development, spanning 4 domains: demographics (age, sex, education level, body mass index); clinical history (hypertension, diabetes mellitus, hyperlipidemia, transient ischemic attack [TIA], smoking status, alcohol intake); laboratory parameters (total cholesterol, low-density lipoprotein cholesterol, high-density lipoprotein cholesterol, triglycerides, fasting glucose, creatinine, fibrinogen, white blood cell count, platelet count); and carotid imaging findings (intima-media thickness [IMT ≥ 1.0 mm] and presence of carotid plaque). These variables, along with their group-wise comparisons between CHD and non-CHD patients, are presented in Table 1.

**Table 1:** Baseline characteristics of patients with and without CHD.

Characteristic		Case group (N = 156)	Control group (N = 286)	*P*-value
*Anthropometric measurements and vital signs*				
Age		67.22 ± 9.14	63.64 ± 9.47	.000
Height (cm)		165.01 ± 6.82	166.74 ± 7.13	.02
Weight (kg)		66.78 ± 9.20	67.19 ± 9.97	.69
BMI		24.49 ± 2.74	24.12 ± 2.94	.19
Educational attainment	Junior high school or below	120 (0.77)	222 (0.78)	
	High school or above	36 (0.23)	64 (0.22)	
Body temperature during onset (°C)		36.52 ± 0.29	36.48 ± 0.23	.96
Respiration during onset (times/min)		18.88 ± 2.10	18.85 ± 1.59	.93
Heart rate during onset (times/min)		75.19 ± 11.27	74.71 ± 10.32	.52
Systolic blood pressure during onset (mm Hg)		146.23 ± 18.58	147.08 ± 19.28	.65
Diastolic blood pressure during onset (mm Hg)		85.95 ± 12.08	88.11 ± 11.87	.25
*Medical history*				
Transient ischemic attack (TIA)		28 (0.18)	26 (0. 09)	.01
Hypertension		104 (0.67)	160 (0.56)	.03
Diabetes		104 (0.67)	193 (0.67)	.86
Hyperlipidemia		18 (0.12)	25 (0.09)	.34
Hyperhomocysteinemia		5 (0.03)	5 (0.02)	.32
*Lifestyle and behaviors*				
Continuous smoking history		33 (0.21)	84 (0.29)	.06
Continuous drinking history		14 (0.09)	43 (0.15)	.07
High-salt diet		35 (0.22)	48 (0.17)	.15
Low-salt diet		60 (0.38)	111 (0.39)	.94
High-fat diet		27 (0.17)	52 (0.18)	.82
Low-fat diet		36 (0.23)	71 (0.25)	.68
High-sugar diet		4 (0.03)	8 (0.03)	.89
Low-sugar diet		25 (0.16)	36 (0.13)	.32
High-protein diet		10 (0.06)	8 (0.03)	.07
Low-protein diet		14 (0.09)	19 (0.07)	.37
Work stress level		153 (0.98)	277 (0.97)	.58
Degree of overwork		146 (0.94)	269 (0.94)	.22
Sleep status		19 (0.12)	38 (0.13)	.64
Exercise		57 (0.37)	111 (0.39)	.64
*Hematological and biochemical indicators*				
RBC (red blood cell count) (×10¹²/L)		4.41 ± 0.50	4.52 ± 0.49	
WBC (white blood cell count) (×10⁹/L)		6.72 ± 1.96	6.84 ± 2.21	.68
PLT (platelet count) (×10⁹/L)		222.28 ± 51.94	218.39 ± 59.30	.42
HGB (hemoglobin concentration) (g/L)		138.92 ± 33.12	141.79 ± 30.64	.01
TC (total cholesterol) (mmol/L)		4.67 ± 1.223	4.6 ± 1.05	.44
Triglyceride (TG) (mmol/L)		1.72 ± 1.08	1.82 ± 1.21	.54
LDL (low-density lipoprotein) (mmol/L)		2.72 ± 1.00	2.7 ± 0.84	.92
HDL (high-density lipoprotein) (mmol/L)		1.22 ± 0.30	1.19 ± 0.31	.34
Apo A1 (apolipoprotein A1) (g/L)		1.22 ± 0.28	1.2 ± 0.23	.51
Apo B (apolipoprotein B) (g/L)		0.93 ± 0.33	0.95 ± 0.45	.55
*Coagulation function*				
PT (plasma prothrombin time) (s)		11.14 ± 2.56	11.53 ± 2.21	.52
FIB (fibrinogen content) (g/L)		3.15 ± 0.77	2.98 ± 0.84	.03
APTT (activated partial thromboplastin time) (s)		28.35 ± 4.17	29.03 ± 4.39	.11
TT (thrombin time) (s)		15.97 ± 3.35	20.24 ± 72.29	.46
*Metabolism and cardiovascular-related*				
HbA1c (glycated hemoglobin) (%)		6.75 ± 1.72	6.76 ± 1.88	.53
Cr (creatinine) (μmol/L)		59.90 ± 17.59	65.70 ± 24.91	.01
UA (uric acid) (μmol/L)		280.00 ± 78.07	287.23 ± 87.04	.38
Hcy (homocysteine) (μmol/L)		15.83 ± 8.53	15.98 ± 11.7	.11

Apo A1 = apolipoprotein A1; Apo B = apolipoprotein B; APTT = activated partial thromboplastin time; BMI = body mass index; CHD = coronary heart disease; Cr = creatinine; FIB = fibrinogen; HbA1c = glycated hemoglobin; Hcy = homocysteine; HDL = high-density lipoprotein; HGB = hemoglobin; LDL = low-density lipoprotein; PLT = platelet count; PT = prothrombin time; RBC = red blood cell count; TC = total cholesterol; TG = triglyceride; TIA = transient ischemic attack; TT = thrombin time; WBC = white blood cell count; UA = uric acid.

Feature selection proceeded in 3 steps. First, univariate LR was applied, and variables with *P* < .05 in Table 1 or strong clinical relevance were retained as potential predictors. Second, these variables were entered into a multivariate LR model to identify independent predictors of CHD, with *P* < .05 as the threshold for significance (Table 2). Third, multicollinearity was assessed using the variance inflation factor (VIF), and variables with VIF >1.5 were excluded to minimize redundancy (see Table S2, Supplemental Digital Content, https://links.lww.com/MD/R154).

**Table 2:** Logistic regression analysis of CHD risk factors.

Factor	OR	SE	Wald	*P*	95% CI	
					Upper limit	Lower limit
Age	1.03	0.02	3.60	.06	1.06	1.00
Height (cm)	0.98	0.02	0.61	.43	1.03	0.94
Hypertension	0.96	0.30	0.02	.89	1.71	0.54
Diabetes	2.19	0.33	5.69	.02	4.18	1.15
Hyperlipidemia	4.98	0.34	21.68	.00	9.79	2.53
Carotid atherosclerotic plaque	4.78	0.30	27.50	.00	8.58	2.66
TIA	2.05	0.36	4.00	.05	4.16	1.01
TC	1.80	0.32	3.32	.07	3.39	0.96
LDL	0.84	0.15	1.30	.25	1.13	0.63
HDL	0.73	0.34	0.85	.36	1.43	0.37
HbAlc	0.57	0.51	1.19	.27	1.55	0.21
RBC	1.10	0.08	1.60	.21	1.28	0.95
HGB	0.57	0.30	3.46	.06	1.03	0.31
FIB	1.00	0.00	0.00	.96	1.01	0.99
Cr	1.04	0.17	0.05	.82	1.45	0.74
Damp-heat constitution	1.00	0.01	0.39	.53	1.01	0.98
Purple-dull tongue	0.34	0.73	2.21	.14	1.41	0.08
Red tongue	1.67	0.28	3.25	.07	2.90	0.96
Fatigue and lassitude	0.55	0.46	1.71	.19	1.35	0.22
Weak voice or feeble cough	1.12	0.30	0.15	.70	2.02	0.63
Loose stools	1.23	0.32	0.43	.51	2.30	0.66
Headache	0.00	14,061.66	0.00	1.00	–	0.00

CI = confidence interval; Cr = creatinine; CHD = coronary heart disease; FIB = fibrinogen; HbA1c = glycated hemoglobin; HDL = high-density lipoprotein; HGB = hemoglobin; OR = odds ratio; LDL = low-density lipoprotein; RBC = red blood cell count; SE = standard error; TC = total cholesterol; TIA = transient ischemic attack.

Following this procedure, 5 independent predictors were retained: age, diabetes mellitus, hyperlipidemia, TIA, and carotid plaque. Carotid IMT, although clinically relevant, was excluded because it did not reach statistical significance in univariate analysis (*P* = .087, Table 1) and demonstrated strong collinearity with carotid plaque (Spearman *R* = 0.76, *P* < .001). All final predictors had VIF values <1.5 (age = 1.03, diabetes = 1.22, hyperlipidemia = 1.14, TIA = 1.10, plaque = 1.30), confirming the absence of problematic multicollinearity.

### 3.2. Model selection rationale

To cover a broad range of ML methodologies and accommodate diverse data assumptions, we selected 7 classifiers from 5 algorithmic groups: linear models, represented by LR, a widely used model in clinical contexts for its interpretability and established utility in cardiovascular prediction; tree-based models, including random forest, XGBoost, and LightGBM, recognized for their capacity to capture complex feature interactions and provide inherent feature importance evaluations; nonparametric classifiers, specifically SVM and KNN, utilized for performance evaluation without explicit parametric assumptions; ensemble learning, demonstrated by a stacking model that amalgamates base learners to explore potential predictive improvements. Due to the limited sample size (n = 442), deep learning techniques were excluded to mitigate the risk of overfitting in intricate models. Additionally, the study prioritized model interpretability, an area where deep neural networks often lack transparency. Survival models like Cox regression were deemed unsuitable given the dataset’s cross-sectional nature. Redundant ensemble methods like AdaBoost were omitted, as XGBoost and LightGBM already embody state-of-the-art gradient boosting techniques, while random forest encompasses bagging-based ensemble capabilities.

### 3.3. Model development and assessment

Seven ML classifiers were developed using the scikit-learn and xgboost libraries: LR, XGBoost, LightGBM, random forest, KNN, SVM, and a stacking ensemble model. The dataset was randomly split into training and testing sets at a 7:3 ratio. Model performance assessment was carried out through 10-fold cross-validation, utilizing metrics such as AUC, accuracy, sensitivity, specificity, F1 score, and average precision. Model calibration was evaluated with calibration plots, and SHAP was used for model interpretability analysis. Clinical applicability was examined through decision curve analysis (DCA). Statistical significance was determined at a *P* *<* .05.

### 3.4. ML pipeline and evaluation metrics

The dataset was randomly partitioned into a 70% training set and a 30% testing set to develop and evaluate models. Seven ML classifiers were employed using the scikit-learn and xgboost libraries: LR, XGBoost, LightGBM, random forest, SVM, KNN, and a stacking ensemble model.

To ensure robust performance assessment, we implemented 10-fold cross-validation on the training dataset. Evaluation of model performance encompassed diverse metrics such as the AUC, accuracy, sensitivity, specificity, F1 score, and average precision. Calibration curves were utilized to gauge the alignment between predicted and actual probabilities of CHD. Enhancing model interpretability involved the integration of SHAP, while assessing clinical relevance entailed the utilization of DCA. Conventional statistical analyses adhered to a significance threshold of *P* < .05.

Hyperparameter optimization and model interpretability were addressed through grid search combined with 10-fold cross-validation to enhance predictive performance across all ML models. Table S1, Supplemental Digital Content, https://links.lww.com/MD/R154 provides detailed information on the critical hyperparameters for each model. To enhance transparency and interpretability, SHAP values were calculated to assess the incremental impact of each feature on prediction outcomes. TreeExplainer was applied to tree-based models like XGBoost, LightGBM, and random forest, while KernelExplainer was used for models without an inherent tree structure. This approach facilitates both global and individualized interpretation of model predictions, thereby bolstering confidence in clinical decision-making.

### 3.5. Addressing class imbalance and optimizing thresholds

Due to the imbalanced class distribution in our dataset (CHD: non-CHD ≈ 1:1.8), we implemented strategies to address its influence on model performance. We adjusted the class_weight parameter in algorithms like LR, random forest, XGBoost, and LightGBM to elevate the cost of misclassifying the minority CHD group. This modification led to enhanced sensitivity and F1 scores without altering the initial data distribution.

In order to preserve the generalizability of the model in real-world clinical settings, we intentionally avoided the use of oversampling methods such as SMOTE, which have the potential to introduce synthetic data points.

In the context of KNN, a method lacking intrinsic support for class weighting, we engaged in threshold optimization utilizing the validation set. Through adjustment of the decision threshold from the standard 0.5 to 0.3, we observed an improvement in the F1 score from 0.405 to 0.423, thereby attaining an improved balance between sensitivity and specificity.

These methodologies collectively enhance the model’s robustness against class imbalance and ensure that the learning process is in line with the crucial task of accurately identifying high-risk CHD patients in clinical settings.

## 4. Results

### 4.1. Baseline characteristics of study participants

A cohort of 442 patients with carotid atherosclerosis was examined, comprising 156 individuals with CHD and 286 without CHD, yielding a CHD prevalence of 35.2%. The CHD cohort demonstrated significantly higher age and a greater prevalence of comorbidities including diabetes mellitus, hyperlipidemia, hypertension, and history of transient ischemic attack (TIA) compared to the non-CHD group. Furthermore, laboratory findings revealed elevated levels of total cholesterol, low-density lipoprotein cholesterol, fibrinogen, and creatinine in the CHD cohort. Additionally, carotid plaque presence was markedly more frequent in CHD patients (*P* < .001). Detailed baseline comparisons are outlined in Table 1.

To evaluate the predictive capacity of carotid plaque, a subgroup analysis was performed based on the stage of carotid atherosclerosis. Patients were divided into 3 categories: individuals with normal IMT without plaque, those with isolated IMT thickening (≥1.0 mm without plaque), and individuals with carotid plaque. The prevalence of CHD was 15.0% in the normal IMT group, 22.5% in the IMT thickening group, and 42.0% in the plaque group, demonstrating a progressive escalation in CHD risk with advancing carotid atherosclerosis stage (see Table S1, Supplemental Digital Content, https://links.lww.com/MD/R154). A gradual rise in CHD prevalence was evident across the spectrum of carotid atherosclerosis: from 15.0% in individuals with normal IMT, to 22.5% in those with isolated IMT thickening, and 42.0% in those with carotid plaque. This pattern is depicted in Figure S2, Supplemental Digital Content, https://links.lww.com/MD/R154.

### 4.2. LR analysis of CHD risk factors

LR analysis was employed to evaluate risk factors for CHD. Initially, 15 variables exhibited notable variances between CHD and non-CHD cohorts in univariate analysis. Subsequently, these variables were incorporated into multivariate LR. The resultant model identified 5 autonomous risk factors significantly associated with CHD in individuals with carotid atherosclerosis: age, diabetes mellitus, hyperlipidemia, history of TIA, and presence of carotid plaque. These 5 variables were designated as pivotal predictive attributes for subsequent ML modeling, as delineated in Table 2.

**Figure 1.**
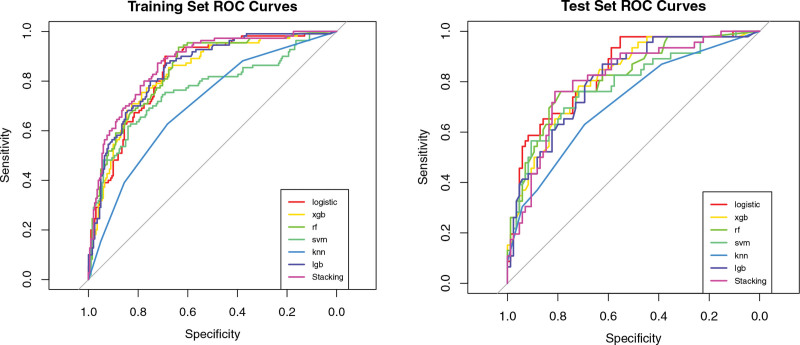
ROC curves of 7 machine learning models. In panel A, the stacking model demonstrated the most superior performance in the training set, achieving the highest AUC of 0.869. Panel B displays the performance on the test set, where logistic regression exhibited the highest AUC of 0.838, suggesting robust generalizability. The ROC curve serves to depict a model’s capacity to differentiate between coronary heart disease (CHD) and non-CHD cases, with a greater AUC signifying enhanced discriminatory ability. AUC = area under the curve, CHD = coronary heart disease, ROC = receiver operating characteristic.

The research identified 5 crucial variables for predictive modeling, outlined in Table 2. Seven ML models were subsequently developed and evaluated for their ability to predict the risk of CHD. Among these models, the stacking ensemble model demonstrated the highest AUC of 0.869 in the training set, accompanied by favorable accuracy (.791), sensitivity (.655), and specificity (.866). In the testing set, LR exhibited the most robust performance, achieving an AUC of 0.838, accuracy of 0.771, sensitivity of 0.587, and specificity of 0.871. Comprehensive performance metrics are provided in Table 3 (training set) and Table 4 (testing set), while Figure 1 depicts the receiver operating characteristic (ROC) curves for all models.

**Table 3:** Performance of CHD risk prediction models in the training set.

Classification model	AUC	Accuracy	Sensitivity	Specificity	F1
Logistic	0.837	0.775	0.627	0.856	0.663
XGboost	0.833	0.788	0.682	0.846	0.694
LightGBM	0.847	0.785	0.664	0.851	0.685
Random forest	0.845	0.765	0.591	0.861	0.640
SVM	0.765	0.759	0.609	0.841	0.641
KNN	0.703	0.691	0.391	0.856	0.473
Stacking	0.869	0.791	0.655	0.866	0.689

AUC = area under the curve; CHD = coronary heart disease; KNN = K-nearest neighbors; SVM = support vector machine.

**Table 4:** Performance of CHD risk prediction models in the test set.

Classification model	AUC	Accuracy	Sensitivity	Specificity	F1
Logistic	0.838	0.771	0.587	0.871	0.643
XGboost	0.815	0.748	0.587	0.835	0.621
LightGBM	0.808	0.748	0.522	0.871	0.593
Random forest	0.817	0.771	0.543	0.894	0.625
SVM	0.786	0.771	0.500	0.918	0.605
KNN	0.718	0.702	0.370	0.882	0.466
Stacking	0.810	0.748	0.587	0.835	0.621

AUC = area under the curve, CHD = coronary heart disease, FRS = Framingham Risk Score, KNN = K-nearest neighbors, SVM = support vector machine.

### 4.3. ML model development and performance

**Figure 2.**
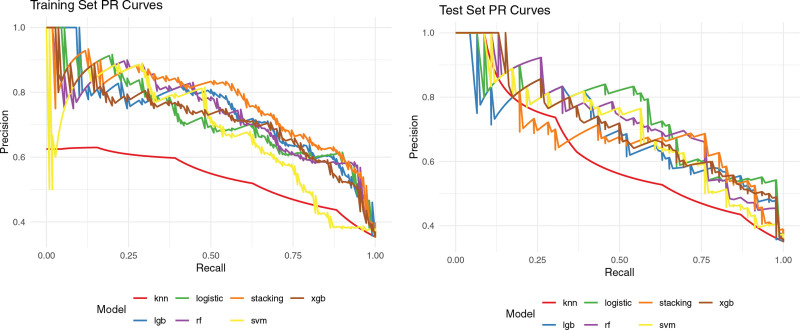
Precision–recall (PR) curves of 7 machine learning models. Panel A displays the precision–recall (PR) curve for the training set, while panel B illustrates the PR curve for the test set. The PR curve is particularly informative in assessing model performance in the presence of class imbalance, where a higher average precision signifies enhanced predictive accuracy. PR = precision–recall.

Seven ML models were trained and assessed to predict CHD risk using 5 selected features. Among these models, the stacking ensemble model achieved the highest AUC of 0.869 in the training set, with favorable accuracy (.791), sensitivity (.655), and specificity (.866). In contrast, LR demonstrated superior performance in the testing set, with an AUC of 0.838, accuracy of 0.771, sensitivity of 0.587, and specificity of 0.871. Detailed performance metrics for the training and testing sets are provided in Tables 3 and 4, respectively. Additionally, Figure 1 depicts the ROC curves for all models, while Figure 2 presents the Precision–Recall curves, emphasizing performance differences in imbalanced settings. To compare our model with traditional risk prediction tools, ROC curves for the FRS and ASCVD estimator were generated separately. Notably, both conventional models exhibited poor discrimination in this high-risk cohort, with AUC values approximating 0.5 (refer to Fig. S1, Supplemental Digital Content, https://links.lww.com/MD/R154), indicating limited predictive value in patients with carotid atherosclerosis.

### 4.4. Model evaluation based on LR

#### 4.4.1. ROC analysis with cross-validation

**Figure 3.**
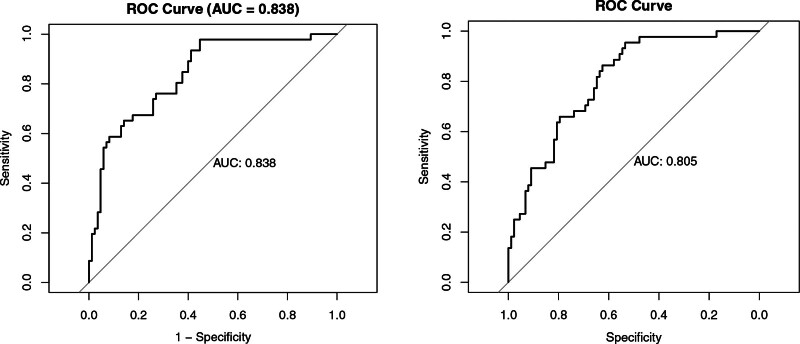
Ten-fold cross-validated ROC curves of the logistic regression model. Panel A displays receiver operating characteristic (ROC) curves generated through 10-fold cross-validation on the training set, with a mean area under the curve (AUC) of 0.805. Panel B exhibits ROC curves on the test set, yielding a mean AUC of 0.838, thereby validating the robustness of the model. AUC = area under the curve, ROC = receiver operating characteristic.

Ten-fold cross-validation confirmed the consistent high performance of the LR model, with a mean AUC of 0.805 in the training set. On the independent testing set, the model achieved an AUC of 0.838, demonstrating strong generalizability. These results support the model’s robust generalizability and discriminative ability, as depicted in Figure 3.

#### 4.4.2. Calibration curve

**Figure 4.**
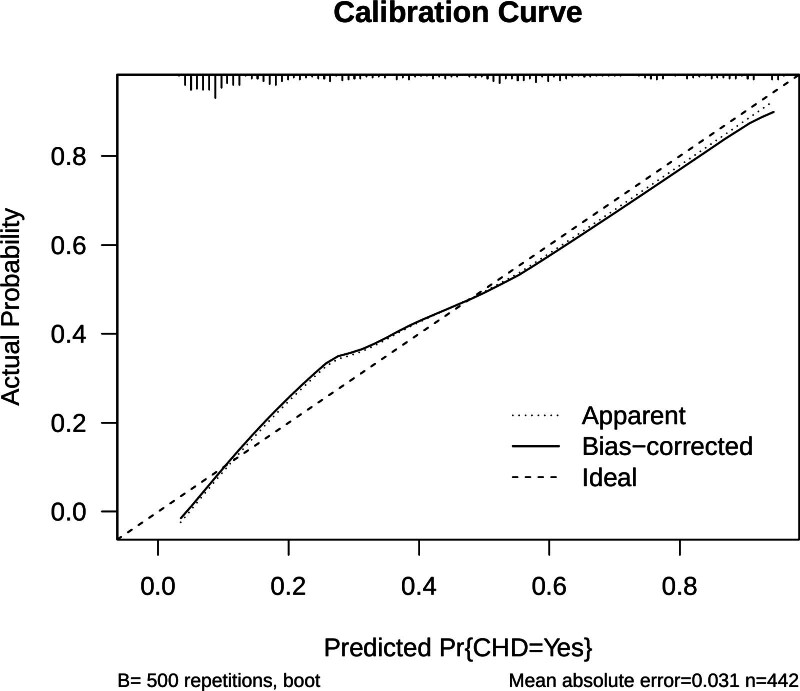
Calibration curve of the logistic regression model. The *X*-axis displays the predicted probabilities of coronary heart disease (CHD), while the *Y*-axis represents the corresponding observed probabilities. The calibration curve exhibits excellent agreement, as the solid line closely tracks the ideal diagonal line, signifying high calibration accuracy. CHD = coronary heart disease.

The calibration plot of the logistic model demonstrated strong concordance between predicted and actual probabilities of CHD, indicating reliable calibration performance (Fig. 4). *Calibration*: the LR model showed strong calibration (Fig. 4), with predicted probabilities closely matching observed risks. This reliability is critical for clinical application and highlights an advantage of our model, as conventional tools such as FRS and ASCVD have not been extensively evaluated for calibration in this high-risk subgroup.

#### 4.4.3. Decision curve analysis

**Figure 5.**
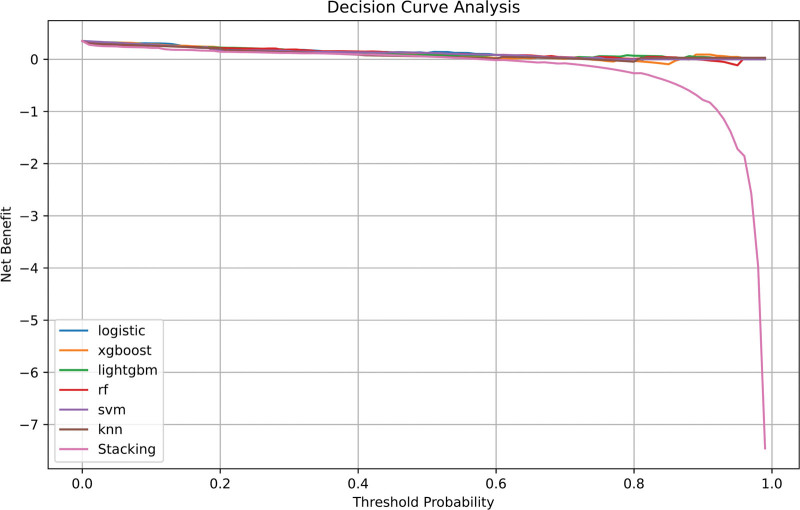
Decision curve analysis (DCA) of 7 models. The *X*-axis denotes the decision threshold probability, while the *Y*-axis illustrates the net clinical benefit. The logistic regression model exhibited optimal net benefit between thresholds of 0.2 and 0.8, suggesting robust clinical utility.

The analysis demonstrated that the logistic model provided the highest net clinical benefit across different threshold probabilities, highlighting its significant clinical value in the early assessment of coronary heart disease risk (Fig. 5).

*Net clinical benefit*: DCA confirmed higher net benefit compared with FRS and ASCVD across a wide range of thresholds (20–80%). For example, at a 30% threshold, our model yielded a net benefit of 0.28 versus 0.05 for FRS and 0.03 for ASCVD (Table 5 and Fig. 5).

**Table 5:** Comparison with traditional cardiovascular risk scores.

Model	AUC	Sensitivity	Specificity
Logistic	0.838	0.587	0.871
FRS	0.524	1.000	0.000
ASCVD	0.498	0.682	0.329

ASCVD = atherosclerotic cardiovascular disease; AUC = area under the receiver operating characteristic curve; CHD = coronary heart disease; FRS = Framingham Risk Score.

### 4.5. SHAP-based interpretability analysis

**Figure 6.**
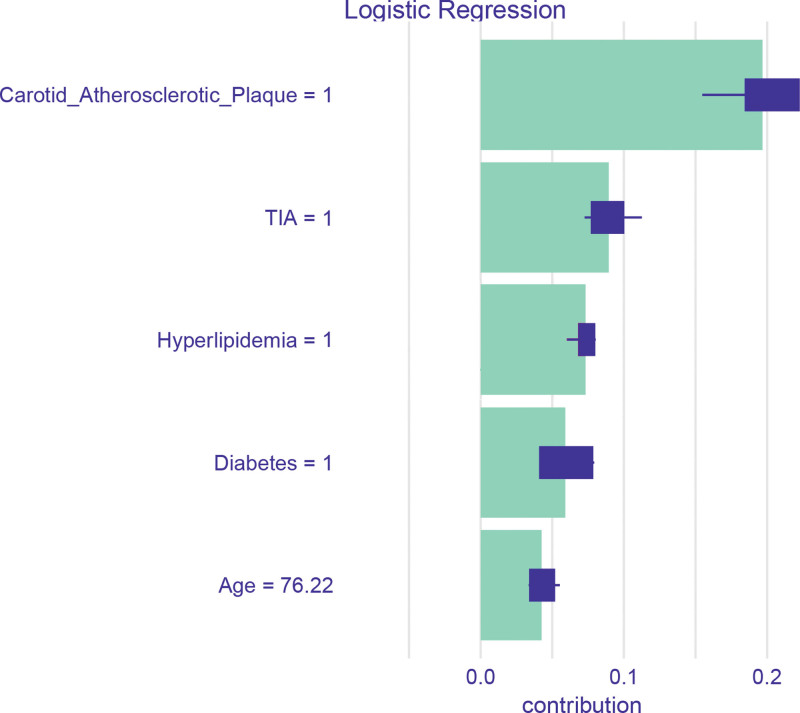
SHAP-based feature importance of the logistic model. SHAP values provide a quantitative assessment of the influence of each feature on the prediction of coronary heart disease (CHD). Among the predictors, carotid plaque exhibited the highest level of influence, with transient ischemic attack (TIA), hyperlipidemia, diabetes, and age also demonstrating positive associations with the risk of CHD. CHD = coronary heart disease, SHAP = Shapley Additive Explanations, TIA = transient ischemic attack.

For enhanced interpretability, we employed SHAP in conjunction with the LR model. The 5 most influential features, as determined by SHAP values, were identified as follows: carotid plaque, history of TIA, hyperlipidemia, diabetes, and age. Notably, carotid plaque exerted the most substantial positive effect on the prediction of CHD risk. Moreover, all 5 features exhibited a positive correlation with the likelihood of CHD occurrence. Detailed visualization of these findings can be found in Figure 6.

### 4.6. Visualization of the CHD risk prediction model

**Figure 7.**
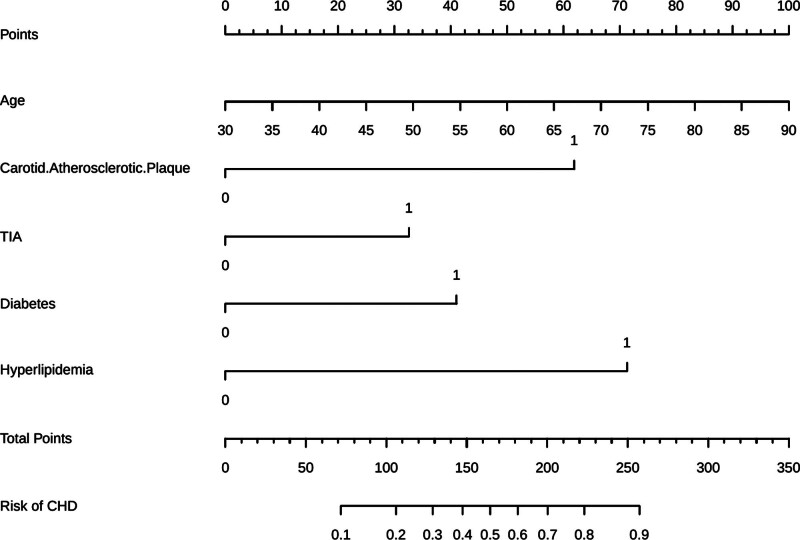
Nomogram for predicting CHD risk in patients with carotid plaque. The nomogram integrates 5 independent predictors: age, diabetes, hyperlipidemia, carotid plaque, and TIA. Usage steps: 1. Position each variable on its respective axis and draw a vertical line to determine its score. 2. Sum individual scores to get a total point value. 3. Map the total score to the CHD risk axis to estimate probability. This tool enables personalized CHD risk assessment and supports early intervention strategies. CHD = coronary heart disease, TIA = transient ischemic attack.

A nomogram was constructed incorporating 5 unique predictors to depict the CHD risk prediction model. Each predictor received a score indicating its relative influence, with total scores corresponding to individualized probabilities of CHD risk. This user-friendly visual tool translates the model into an accessible format for frontline clinicians, enabling accurate and individualized risk estimation for patients with carotid atherosclerosis (Fig. 7).

## 5. Discussion

This study presents a novel ML model aimed at predicting the probability of CHD in individuals with early-stage carotid atherosclerosis, offering a practical tool for personalized cardiovascular risk assessment. Among the 5 key predictors (age, diabetes mellitus, hyperlipidemia, transient ischemic attack [TIA], and carotid plaque) the SHAP analysis identified carotid plaque as the most significant contributor to CHD risk. While the study’s inclusion criteria focused on carotid atherosclerosis, this encompassed a spectrum ranging from isolated IMT to advanced plaque formation.^[13]^ Notably, within this spectrum, the presence of plaque emerged as the primary indicator of increased CHD risk.

Initially included in the definition of carotid atherosclerosis, IMT was subsequently excluded from the final model due to its lack of statistical significance in univariate analysis (*P* > .05) and its substantial collinearity with plaque presence (Spearman *R* = 0.76, *P* *<* .001). Stratified analysis reaffirmed these findings: the prevalence of CHD was significantly higher in individuals with carotid plaque (42.0%) compared to those with only IMT thickening (22.5%) or normal IMT (15.0%). These results suggest that while IMT reflects early vascular remodeling, the presence of plaque signifies a more advanced and unstable atherosclerotic phenotype. These observations align with the Atherosclerosis Risk in Communities and Tromsø studies, which emphasized a robust association between plaque burden and future coronary events.

Carotid plaque reflects the systemic atherosclerotic burden and mirrors coronary artery pathology, sharing common mechanisms such as endothelial dysfunction, lipid accumulation, oxidative stress, and chronic inflammation across vascular regions. Studies have shown that the presence of carotid plaque is a more robust predictor of future coronary events than IMT alone. For instance, data from the Atherosclerosis Risk in Communities study demonstrated that carotid plaque, rather than IMT, independently correlated with CHD development after adjusting for traditional risk factors.^[14,15]^ This heightened predictive capacity is attributed to the advanced and precarious vascular restructuring indicated by plaque formation, involving processes like necrotic core formation, fibrous cap thinning, and intraplaque hemorrhage (features akin to coronary plaque rupture).^[16]^ Previous research has also established strong links between carotid plaque burden and coronary artery calcium scores or confirmed coronary lesions, underscoring carotid plaque as not only a local vascular damage marker but also a systemic indicator of elevated coronary risk.^[17]^

The inclusion of transient ischemic attack (TIA) significantly enhances the prognostic value of the model by capturing the precursor role of TIA in vascular instability and microembolization, often antecedent to cerebrovascular and cardiovascular events. When TIA is combined with metabolic comorbidities such as diabetes and hyperlipidemia, which accelerate plaque progression and instability, the model comprehensively assesses cumulative vascular damage over time. LR was selected for its optimal balance between predictive accuracy and clinical interpretability, achieving an AUC of 0.838 in the validation cohort. In contrast to established risk assessment tools like the FRS and ASCVD score, recommended in the 2019 ACC/AHA Primary Prevention Guideline,^[5]^ which exhibited AUCs near 0.5 in this high-risk population, our model demonstrates superior discriminatory performance. This suggests that conventional models may be insufficient for risk stratification in individuals with asymptomatic carotid artery disease.

This research highlights the unique effectiveness of SHAP analysis in improving the interpretability and clinical significance of ML models, surpassing traditional performance metrics.^[18]^ SHAP values not only evaluate the general significance of features but also provide individualized explanations for risk forecasts at the patient level. This capacity advances a precision medicine approach, enabling healthcare providers to tailor interventions according to distinct risk factors, whether stemming from metabolic dysregulation, neurological history, or vascular imaging outcomes. In addition to enhancing interpretability, it is equally important to translate predictive models into practical tools that can be readily applied in daily clinical practice. Furthermore, developing a nomogram based on LR coefficients provides a practical visual tool that can be easily used at the bedside. Risk assessment instruments created using this approach can be smoothly incorporated into electronic health records or utilized in mobile apps to support well-informed decision-making, especially in primary care or community screening settings.

Despite the promising performance of our model, several limitations require attention. Firstly, this study’s single-center design may restrict its generalizability to more diverse populations with varying demographic and clinical characteristics. Variations in atherosclerotic patterns and clinical practices across regions could influence the model’s performance, underscoring the need for external validation using multicenter datasets. Secondly, the assessment of carotid plaque relied on imaging modalities (Doppler ultrasound or CTA) selected based on clinical availability, potentially introducing interpretation bias. Future research should adopt standardized imaging protocols to ensure measurement consistency. Thirdly, despite a class imbalance between the CHD and non-CHD groups, we managed this by adjusting class weights and fine-tuning thresholds rather than resorting to oversampling, thereby maintaining data integrity. Finally, unlike traditional risk scores (e.g., Framingham, ASCVD) that estimate long-term CHD incidence, our model focused on risk stratification of existing CHD at the time of evaluation. However, the lack of prospective follow-up data limited time-based prediction (e.g., 1-year or 10-year risk). Future longitudinal studies are needed to extend this approach into survival models and enable direct comparison with conventional time-bound risk scores. These limitations highlight the necessity for prospective validation and real-world implementation to confirm the model’s clinical utility for early CHD risk stratification in patients with carotid plaque.

This research introduces a ML algorithm tailored to forecast the likelihood of CHD in patients with carotid atherosclerosis. The model integrates key demographic, metabolic, and imaging variables, demonstrating robust discriminatory and calibration performance. Notably, the presence of carotid plaque emerges as the most influential predictor, highlighting its clinical relevance in identifying high-risk individuals. By training and validating the model on a cohort encompassing various stages of carotid atherosclerosis, its applicability primarily targets individuals with confirmed carotid plaque, representing an advanced and actionable phase of subclinical disease. This model holds promise in assisting healthcare providers with early risk evaluation and guiding targeted preventive strategies within this high-risk subgroup. Furthermore, it could function as a practical screening tool in outpatient and primary care settings, aiding clinicians in prompt decision-making regarding cardiovascular interventions.

## Author contributions

**Conceptualization:** Mengke Lyu.

**Data curation:** Lei Zhang, Mengke Lyu, Mingyuan Du, Yizhuo Li, Haifeng Yan, Xiaohui Li, Wenshuang Niu, Lizhi Pang.

**Methodology:** Mengke Lyu.

**Resources:** Lei Zhang.

**Writing – original draft:** Lei Zhang, Mengke Lyu.

**Writing – review & editing:** Mengke Lyu.

## Supplementary Material


