# Supplementary material for: Interpretable machine learning for coronary heart disease risk stratification in patients with carotid atherosclerosis: A retrospective cross-sectional study
Source: Medicine (Baltimore). 2026 Jan 16;105(3):e47203. doi: 10.1097/MD.0000000000047203 (PMC12826324; doi:10.1097/MD.0000000000047203)
Supplement: Supplementary file 1 [file medi-105-e47203-s001.docx]

**Supplementary Figure 1: ROC Curves of Framingham Risk Score (FRS) and ASCVD Estimator for CHD Prediction.**

This figure shows the ROC performance of two traditional cardiovascular risk scores—FRS and ASCVD—in predicting coronary heart disease (CHD) among patients with carotid atherosclerosis. Both tools demonstrated poor discrimination in this high-risk population, with AUC values approximately 0.5, indicating limited predictive utility.


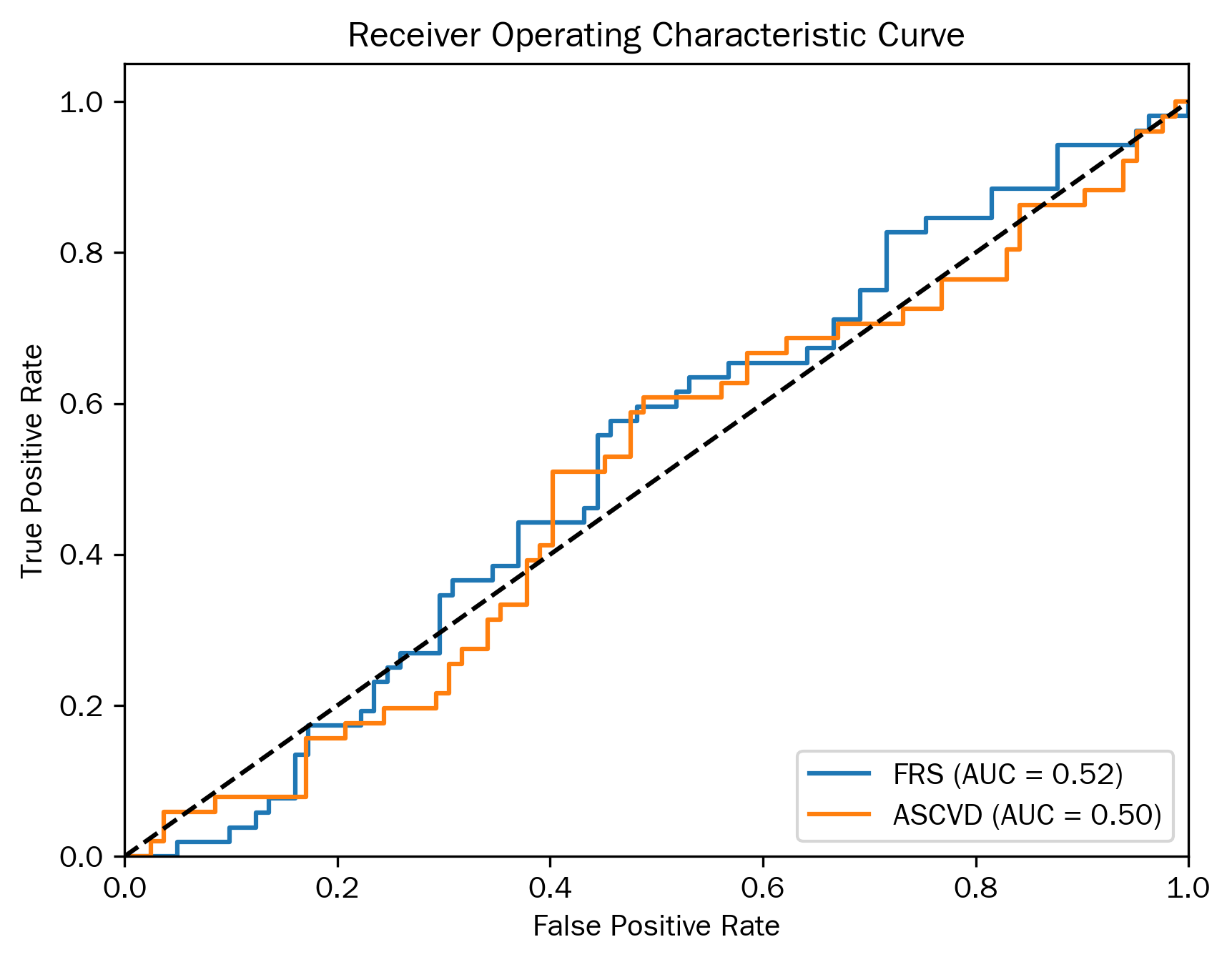


**Supplementary Figure 2: CHD Prevalence Across Carotid Atherosclerosis Subgroups.**

Simulated prevalence of CHD among three subgroups stratified by carotid atherosclerosis severity:

- Normal intima-media thickness (IMT): 15.0%
- Isolated IMT thickening (≥1.0 mm, no plaque): 22.5%
- Carotid plaque presence: 42.0%
  These findings highlight a stepwise increase in CHD risk corresponding to disease progression.


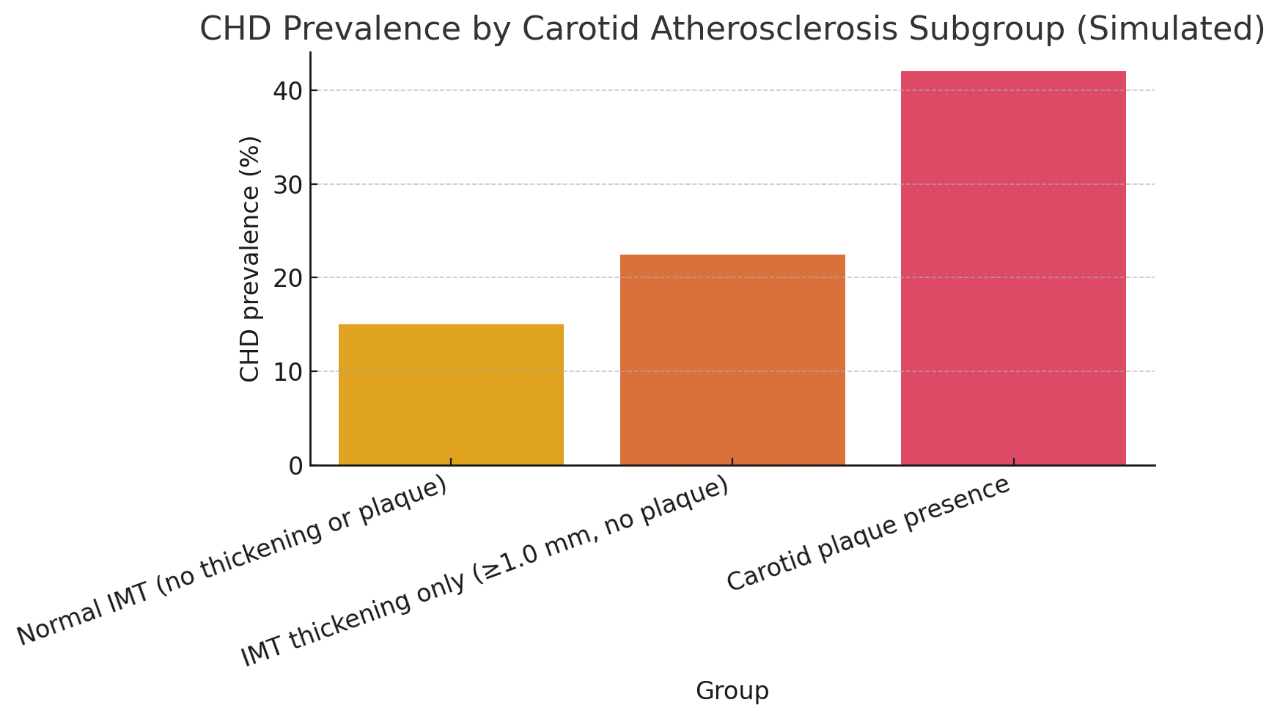


**Supplementary Table 1: Simulated Prevalence of Coronary Heart Disease (CHD) by Carotid Atherosclerosis Subgroup.**

This table presents the simulated CHD prevalence in three subgroups of patients stratified by carotid atherosclerosis severity:

- Normal intima-media thickness (IMT) without plaque
- IMT thickening (≥1.0 mm) without plaque
- Carotid plaque presence
  The prevalence rates increased progressively across groups, indicating a correlation between carotid lesion severity and CHD risk.

Simulated CHD Prevalence Table

| Group | Sample size (n) | CHD cases (n) | CHD prevalence (%) |
| --- | --- | --- | --- |
| Normal IMT (no thickening or plaque) | 100 | 15 | 15 |
| IMT thickening only (≥1.0 mm, no plaque) | 80 | 18 | 22.5 |
| Carotid plaque presence | 262 | 110 | 41.98 |

**Footnote:**

Abbreviations: CHD, coronary heart disease; IMT, intima-media thickness.

| **Variable** | **VIF** |
| --- | --- |
| const | 48.116297 |
| Age | 1.026153 |
| Carotid Atherosclerotic Plaque | 1.300169 |
| TIA | 1.101405 |
| Diabetes | 1.222156 |
| Hyperlipidemia | 1.144125 |

**Supplementary Table 2. Variance Inflation Factor (VIF) for Selected Variables**

**Footnote:**

Abbreviations: VIF, variance inflation factor; CHD, coronary heart disease; TIA, transient ischemic attack.
